# Supplementary material for: Preventing Childhood Obesity in Primary Schools: A Realist Review from UK Perspective
Source: Int J Environ Res Public Health. 2021 Dec 20;18(24):13395. doi: 10.3390/ijerph182413395 (PMC8702173; doi:10.3390/ijerph182413395)
Supplement: Supplementary file 1 [file ijerph-18-13395-s001.zip › ijerph-1469007-supplementary.pdf]

Supporting information

Preventing Childhood Obesity in Primary Schools: A Realist Review from UK Perspective

Authors: Sharea Ijaz, James Nobles, Laura Johnson, Theresa Moore, Jelena Savovic and Russell Jago

Section S1 programme theory development

V2: Adapted based upon stakeholder survey and initial piloting

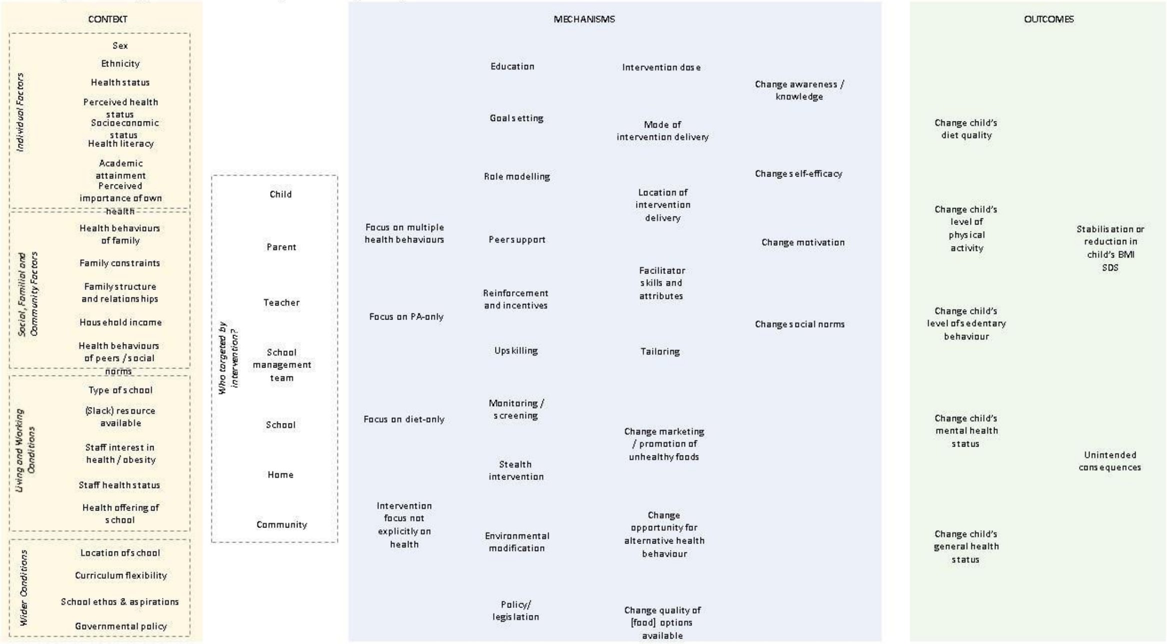

V3: Refinement after further testing

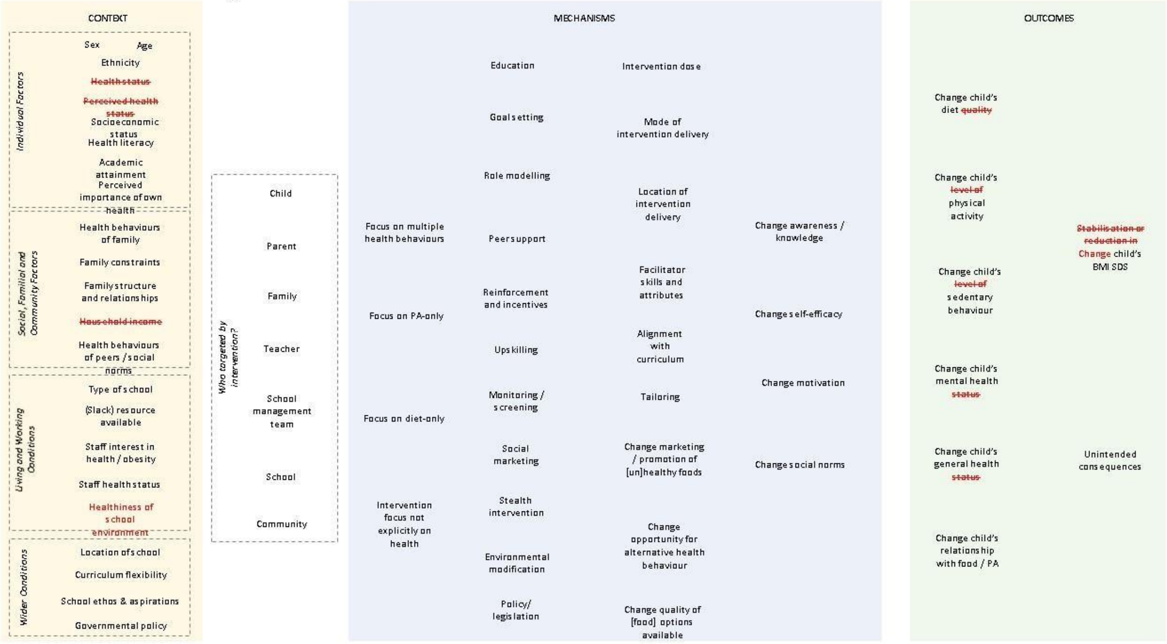

V4: Refinement during data extraction

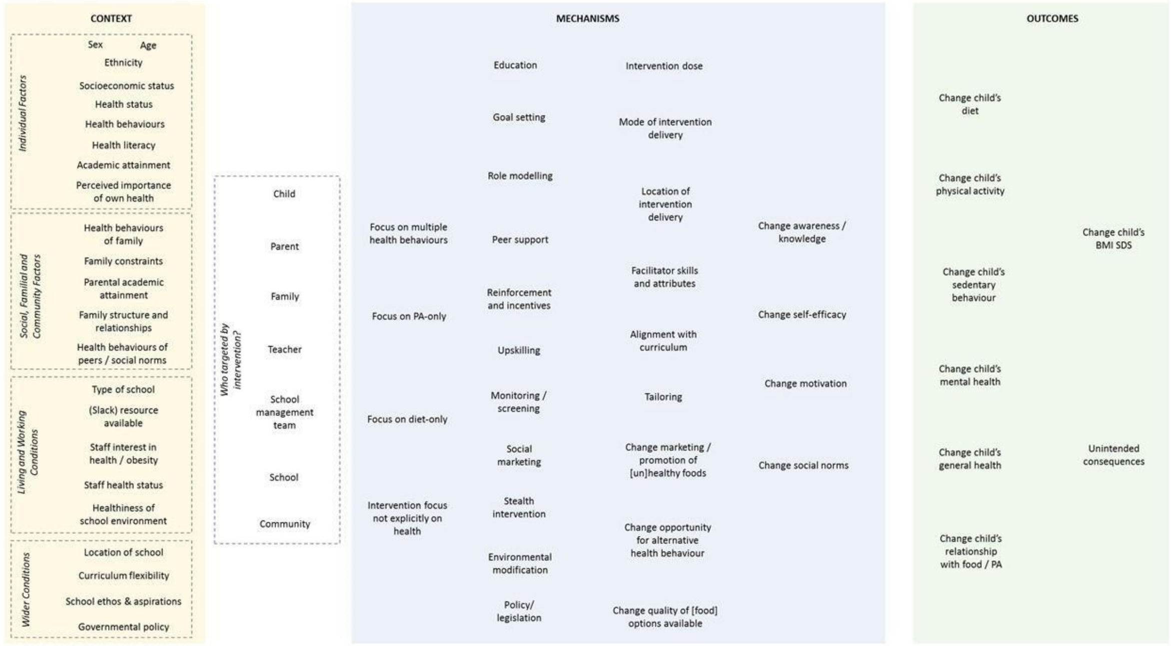

V5: Further refinement during data extraction

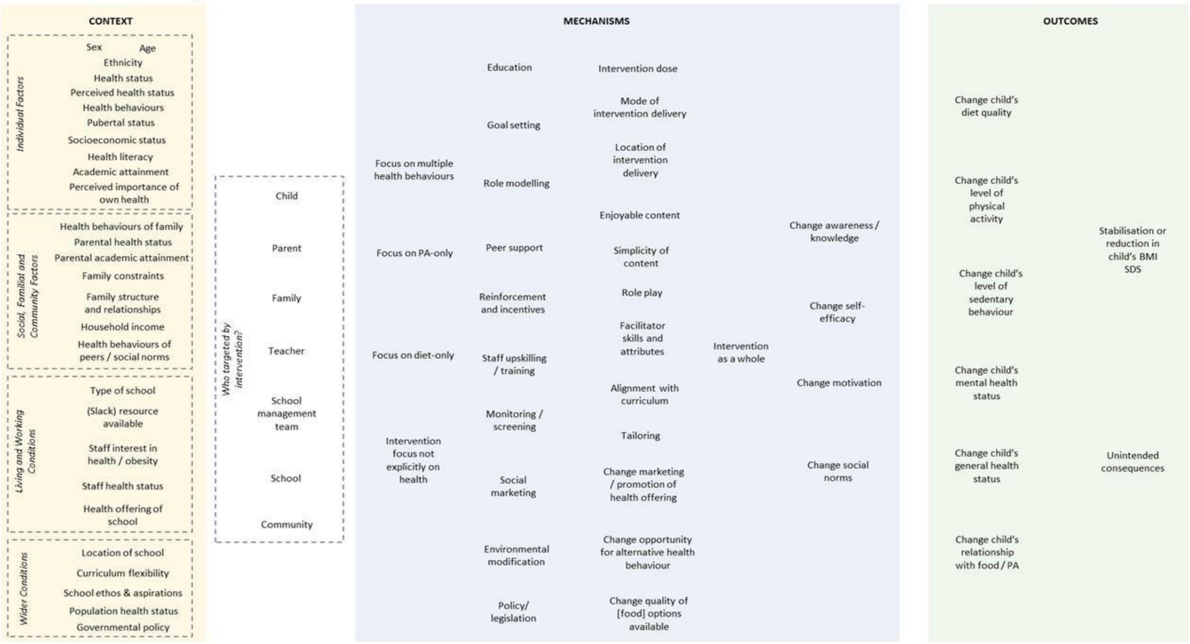

V6: Final

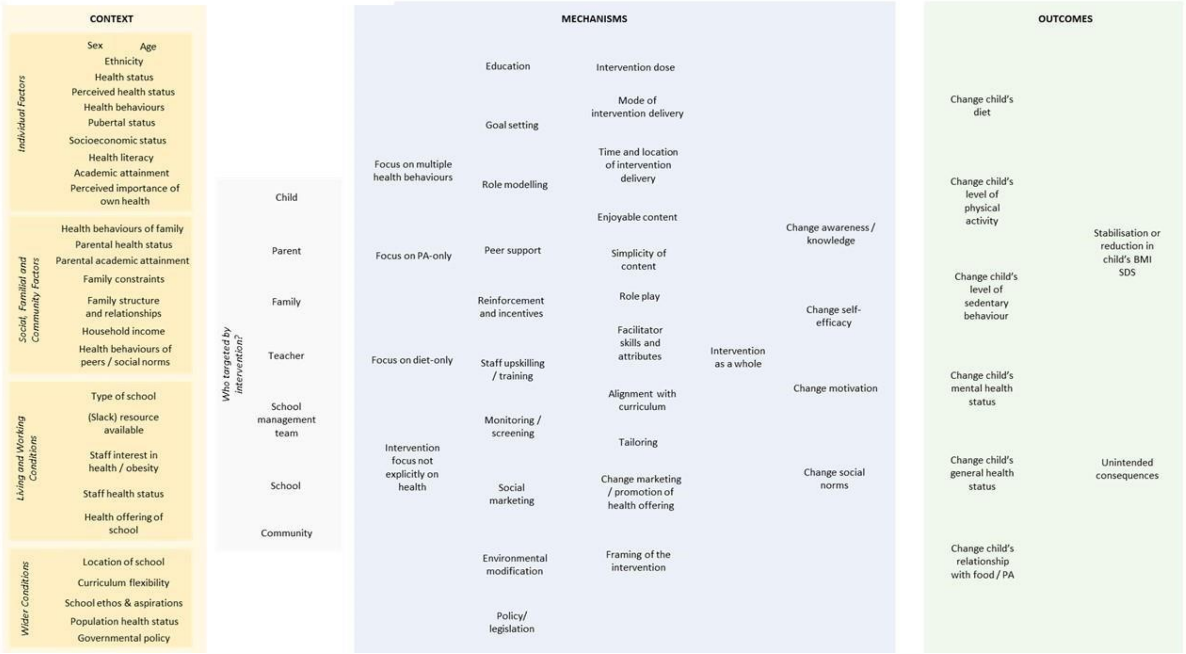
CONTEXT
Individual Factors
Sex
Age
Ethnicity
Health status
Perceived health status
Health behaviours
Pubertal status
Socioeconomic status
Health literacy
Academic attainment
Perceived importance of own health
Social, Familial and Community Factors
Health behaviours of family
Parental health status
Parental academic attainment
Family constraints
Family structure and relationships
Household income
Health behaviours of peers / social norms
Living and Working Conditions
Type of school
(Slack) resource available
Staff interest in health / obesity
Staff health status
Health offering of school
Wider Conditions
Location of school
Curriculum flexibility
School ethos & aspirations
Population health status
Governmental policy
Who targeted by intervention?
Child
Parent
Family
Teacher
School management team
School
Community
MECHANISMS
Focus on multiple health behaviours
Focus on PA-only
Focus on diet-only
Intervention focus not explicitly on health
Education
Goal setting
Role modelling
Peer support
Reinforcement and incentives
Staff upskilling / training
Monitoring / screening
Social marketing
Environmental modification
Policy/ legislation
Intervention dose
Mode of intervention delivery
Time and location of intervention delivery
Enjoyable content
Simplicity of content
Role play
Facilitator skills and attributes
Alignment with curriculum
Tailoring
Change marketing / promotion of health offering
Framing of the intervention
Intervention as a whole
Change awareness / knowledge
Change self-efficacy
Change motivation
Change social norms
OUTCOMES
Change child's diet
Change child's level of physical activity
Change child's level of sedentary behaviour
Change child's mental health status
Change child's general health status
Change child's relationship with food / PA
Stabilisation or reduction in child's BMI SDS
Unintended consequences

Section S2 data extraction template

**DE item**	**Comments**	**Text copied in**
First author and date of publication of document(s) mark which paper the data came from below (e.g. (James 2004) or (James 2007))		
Study location city (country), add text where it is of relevance to context		
Study design(s) (RCT, Mixed method, qualitative, etc.), add text if they justify the methods		
Duration of intervention (of study) in months		
Duration of follow up (for outcome assessment) in months		
Timing (during/ before/ after school)		
Aim (briefly in authors' own words if possible)		
Incentive provided for participation: Monetary or other(s)		
Cost of intervention reported (convert to GBP 2019 value)		
Context: list all reported by matching with the program theory diagram - then also add any additional ones they refer to that are not in our theory diagram but should be<br><br>(N = Number of contexts identified)		
Intervention characteristics (target group, providers, time, duration, intensity, components in authors' words- copy text in)		
Mechanisms triggered-match with program theory diagram and list all that fit- then also add any additional ones they refer to that are not in our theory diagram but should be<br><br>(N= Number of mechanisms identified)		
Outcomes – keep this simple and list only those matched with the program theory diagram		

(N =Number of outcomes identified)		
Conclusions of the authors in their words		
Relevance: data (text sections) within a study that show relevance to our theory (for either development or testing),		
Rigour: study's methods of data collection and analysis and whether we can trust what their claims are		

Section S3 example of data extraction

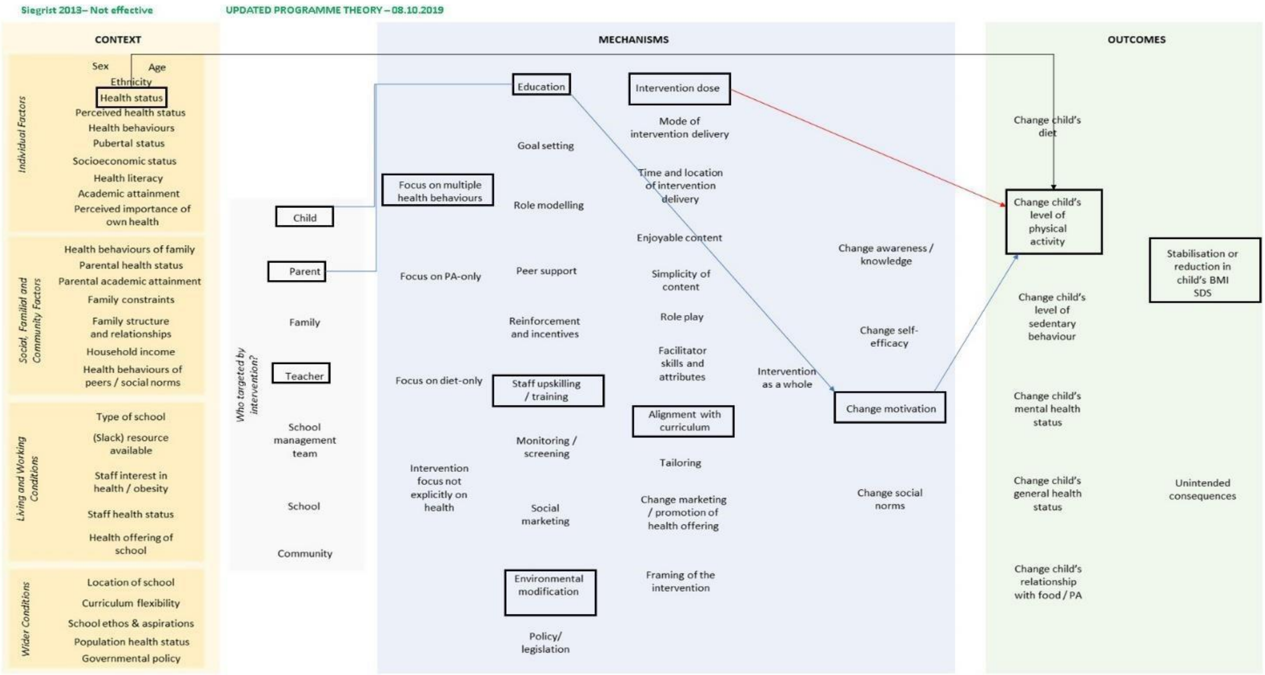

Context:

Health status-overweight kids had improved PA after intervention: Overweight and obese children showed lower baseline fitness levels. After 1 year, they had significantly improved in two test items (goal throwing and jump and reach) and slightly increased their total score in contrast to a decrease in the controls. *Siegrist 2013 Page 329 para 2* **Mechanisms: Education /aligned with curriculum/ environmentmodification/ multiple behaviours:** The focus of the multifaceted JuvenTUMintervention was on directly educating and encouraging children, teachers, and parents to live active and healthy lifestyles. Additionally, school environmental settings (e.g. the physical environment, organization of school breaks, playing during school time, and sports facilities) were altered to promote more physical activity. These changes were designed to increase physical movement, promote healthier food availability and choices (more vegetables and fruits and less energy-dense food), and reduce media consumption (for further details about the program, please visit our website http://www.juventum.med.tum.de). *Siegrist 2013-page 325 para 2-3*

Dose (duration) not enough for PA change Although physical activity remained unchanged in the control group, no significant difference between both groups was found after 1 year. It is possible that the sensitivity of physical activity questionnaires is inadequate to allow for the detection of significant group differences or that the intervention time was to short (10 lessons over a period of 1 year). These questions are being investigated in an ongoing longer term intervention. *Siegrist 2013 page 328 para 4* **.**

That’s not reliable data then is it? don’t buy this argument though…because they have used valid PA measure: https://www.ncbi.nlm.nih.gov/pubmed/11343497.

environment modification> change in quality of food / changed opportunity for alt health behaviour: Additionally, measures were taken to improve the quality of food sold at school snack bars and school stores as well as to arrange the classrooms, halls, and playgrounds in a way to promote more physical activity. All teachers of the intervention schools (IS) took part in these trainings.*Siegrist2013-page 325 para 3-6*

Education > increased motivation and competence? Change in PA: Successful learning experiences in skill development are particularly important in overweight children to enhance bildren’s pereption of ompetene and motivation, whib in turn maximizes participation in physical activity.*Siegrist2013 Page 329 para 2*

Teacher training/ upskilling: Three teacher trainings (9 h total) were conducted with the objective of increasing their students’ physial activity during lessons and breaks and improving physical education within their schools.*Siegrist 2013-page 325 para 6*

Parents targeted with education: Parents participated in two training sessions in which they were given a program overview and practical instruction about health issues (3 h total). They were informed about the development and course of the intervention program, received health-related journals, participated in practical instruction based on increasing motivation to spend more time being active with their children, and were asked to improve health behaviors(e.g. making healthy food choices and less media consumption) with their family. 30% participated.*Siegrist 2013-page 325 para 5*

Rigour comment: issues with their arguments-see above. Plus, they say they lack control for dietary behaviours in the study. But they added info on diet in education as part of the intervention – if not then why add it? Randomisation was unclear; rest of the RoB items were at low risk and analysis appropriate.

Section S4 cost and sustainability data

**Study and country**	**Cost information**	**2020 cost in GBP (https://eppi.ioe.ac.uk/costconversion/ default.aspx)**
Marcus 2009 Sweden	The intervention was aimed to be financed within the resources of the ordinary school budget.	NA

Khan 2014 USA	Funding figure for 2008 on NIH website states $341,654 for the school year.	£1316.71 per child / year Assuming this is the total cost and divide by number of participants (220) = $1553 per Child per year.
Kipping 2014 UK	The main cost drivers for the intervention were the claims by the schools for replacement teachers needed to cover the teachers' attendance at the training days (£5.00 per pupil, £5095.00 in total); the trainers' fees (£2.00 per pupil, £2166.00 in total); the time spent by the research staff organising and attending the training days (£2.00 per pupil, £2492.00 in total); and the printing costs of the materials for the AFLY5 lessons and the homework (£6.00 per pupil, £6694.00 in total). We estimated the opportunity cost of implementing AFLY5 in schools (i.e. the cost of teaching AFLY5 minus the cost of teaching the usual curriculum based on data from schools in the control arm) to be £0.05 per pupil. The costs varied by school, ranging from approximately £13.00 to £36.00 per pupil. The variations between the schools were driven by the costs of the teachers' attendance at the training days. The cost–consequence analysis showed that, for the three secondary outcomes that were affected by the intervention, it cost £18 per child (£18,944 in total) to reduce self-reported time spent on screen viewing at the weekend by 20.86 minutes, self-reported consumption of snacks by 0.22 snacks per day and self-reported consumption of high-energy drinks by 0.26 servings per day. From the teachers' perspective, teachers spent, on average, more time travelling to the training day venue than they usually spent travelling to school; this equated to an additional 0.68 minutes' travel time per pupil, generating an extra cost of £0.19 per child (£206 in total). *page 43 of PHR full report 2016* Results of the economic analysis showed that the cost per child from a school and provider perspective of implementing the intervention was £18 per pupil (£18,944 in total). *PHR full report discussion page 64 para 3*	£20.03 per child/ 8 month
Damsgaard 2014 Denmark	NR. **Comment: They say in protocol they measured costs but these are not reported in any of the outcome papers**	NA

Meng 2013 China	social perspective- For year 2013-all costs reported in table 3 and 4; ICERS in table 5<br>The intervention costs per child in combined intervention group was RMB182.4 ($26.8), which was 2.4 times higher than that in the nutrition intervention (RMB52.8, $7.8) or in the PA intervention (RMB52.3, $7.7). page 5 para 3<br>conversion to GBP 2019: costs per child in combined intervention group= £24.1 cost per child nutrition intervention= £ 7.04 cost per child PA intervention= £ 6.97	Combined intervention: £21.05 per child/ 6 month Diet intervention: £6.13 per child/ 6 month PA intervention: £6.05 per child/ 6 month
Mucklebauer 2010 Germany	In our study, the initial costs per water fountain were ~2500 euros and the long-term costs per enrolled child were _13 euros per year. The educational intervention was presented by the teachers; therefore, no additive costs emerged.<br>Muckelbauer 2009—discussion)<br>The schools were provided the running costs of the water fountains (about 800 Euros/year) by the study budget in the intervention period and the following year and had to pay the costs themselves afterwards. Muckelbauer 2009a page 852- [para 8)<br>11 out of 17 schools succeeded in keeping the fountains (Table 1). Headmasters of the schools that kept the fountains reported that maintaining costs were paid by fees from parents (*n* = 6), bounties (*n* = 3), school fund (*n* = 4), and other school-related associations (*n* = 3), solely or in combination. (Muckelbauer 2009a page 853- para 5)<br>**Comment: These are values for year 2007.**<br>**Unclear how educational intervention had 'no additive' cost- at least materials would have a cost.**	Cost of fountain: £2643.85<br>Cost of yearly maintenance: £846.03<br>Cost for 2 years: £3,489.88<br><br>For 2500 children the cost per child for first 2 years: £1.39<br><br>For 250 children the cost per child for first 2 years: £13.9
	**Long term not defined**<br>**Cost of bottles not reported**	
Rush 2012 NewZealand	The programme is cost-effective, the main costs are the salaries of the Energizers and team leader and the travel required to move between schools. We calculate that the average cost of the intervention for each child, each year, is less than $40 New Zealand and this could be improved by further efficiencies. *Rush 2012 page 585 para 1*	£22 per child/ year
Grydeland 2014 Norway	NR.<br>**Comment: In protocol they say they were collecting cost data for CE analysis and in the final paper they say it didn't cost much as teachers were the main providers.**	NA
Paineau 2008	Family dietary coaching has an individual cost of around 1 €/d/person (US $1.42/d/person), which should be compared with the cost implications of obesity for health care and society.<br>**Comment: Unclear if it is their cost or taken from somewhere else.**	NA

Study and country	Supporting text from study	Key facilitators

Flexibility considerations		
Li (2010)<br>China	*Exercise intensity varied by grade levels because of the different activities involved and the different rhythm and extent of the same activity. Students in lower grades were more active than those in higher grades. Students in grade 3 were most active, with the highest energy expenditure and intensity of a Happy 10 session.[…] The program provided a variety of safe, moderate, age-, and space-appropriate exercises . Teaching materials included activity cards, video demonstrations, tracking posters, and stickers. Each activity card introduced one exercise and explained how to perform it. The videos showed students from the pilot study performing the activities. Teachers could either demonstrate the activity or show it on a video. The tracking poster and stickers were used to illustrate the progress of each class. Students, teachers and parents were encouraged to develop new activity models, so did the program staffs. Many new programs, much more than that directly from TAKE 10!, were developed, such as*<br>*"Story in zoo"; "story in farm"; "who is wearing yellow today"; "time like a colt"; "happy and health"; "little frog". –* pg 181	1. Provide teachers with several options for how to deliver elements of the intervention.<br>2. Teachers able to adapt the intervention based on their knowledge of the pupils ability and preferences.<br>3. Co-produce materials with children, families and teachers (for sustainability).
Rosario (2012)<br>Portugal	*Our approach was to standardize recommendations to teachers, allowing them enough flexibility to create interactive interventions and pedagogic instruments to be used with children. This is contrary to previous school-based interventions that have used tight controls to ensure uniform implementation but required frequent staff training and ongoing supports. –* pg 1364	1. Ensure recommendations for teachers are consistent, however allow flexibility in how the information is delivered.
Sustainability considerations		
Marcus (2009)<br>Sweden	*The programme was designed to be an integrated, sustainable part of the ordinary school curriculum, possible to maintain within the ordinary school budget. –* pg. 415	1. Embedding the intervention within the curriculum.
Muckelbauer (2009)<br>Germany	*The schools were provided the running costs of the water fountains (about 800 Euros/year) by the study budget in the intervention period and the following year and had to pay the costs themselves afterwards. –* pg. 852<br>*11 out of 17 schools succeeded in keeping the fountains (Table 1). Headmasters of the schools that kept the fountains reported that maintaining costs were paid by fees from parents (n = 6), bounties (n = 3), school fund (n = 4), and other school-related associations (n = 3), solely or in combination. -* pg. 853 *A widespread transfer of our programme is favoured by the fact that implementation was exclusively based on school staff. This independency of external support enhances the practicability and sustainability of the programme. However, external financial support may increase the number of schools maintaining the fountains when the initial installation of the fountains has been*	1. Ensure educational components can be delivered by school staff.<br>2. Target intervention at school staff rather than children. Reduces need for external support.<br>3. Ensure financial cost of maintaining intervention is low and available (€800).
	managed. The fact that even in deprived districts, the vast majority of schools were willing and able to find a financing for the fountains is of particular significance – pg 856	
Kipping (2014)<br>UK	*Although the quantity of lessons and homework assignments delivered was high, the difficulties of incorporating some of the AFLY5 materials into more technologically advanced and interactive current teaching practice, coupled with pressure on teachers' time and a need to adapt the materials to suit students' differing abilities and ensure their engagement, resulted in mixed enthusiasm for AFLY5.–* pg. 70	1. Ensure intervention resources are delivered in a similar format to existing school resources.<br>2. Ensure resources do not require much modification by teachers.
Gutin (2008)<br>USA	*Institutionalization of innovative programs requires that the program be built on existing infrastructure and resources. MCG FitKid was built on the infrastructure in the schools (i.e., school teachers and paraprofessionals, facilities, and transportation system), which increases the potential for success of the program and the likelihood of adoption in other schools and communities. -* Yin 2005, pg. 2160	1. Limit the need for additional infrastructure and resource when implementing a school-based intervention.

Grydeland (2014) Norway	*The results of this intervention study are important to public health, as feasibility and sustainability were high priorities when designing the intervention. This has been recommended in previous studies and reviews [6,8,37]. Although comprehensive, the intervention components were designed to be able to fit into current school curricula without substantial extra costs. With limited instructions and material provided by the study group, teachers were key deliverers of the intervention components. No extra personnel or costly material are needed to carry out such components in the current school system, and all components could easily be incorporated into existing curricula for this age group. –* pg. 11	1. Align intervention content with the school curriculum.<br>2. Ensure intervention can be implemented for limited additional cost.<br>3. Intervention content should be simple and easy for teachers to deliver.
Fairclough (2013) UK	*Interventions that can be implemented by school personnel in 'real life' conditions (i.e., without re searcher support and resources) are advocated [15], as these are less costly [13], and are more likely to be integrated within existing curricula and sustained over time. –* pg. 2	1. Ensure intervention can be delivered without additional resource from study team.<br>2. Align intervention content within the curriculum.
Stakeholder involvement considerations		
Foster (2008) USA	*Each school formed a Nutrition Advisory Group to guide the assessment. Teams included administrators, teachers, nurses, coaches, and parents. After completing ratings on healthy eating and physical activity, schools developed an action plan for change. –* pg. 795	1. Create diverse stakeholder advisory groups and include in intervention design and delivery.
Fairclough (2013) UK	*The intervention design and content were informed by formative work conducted with parents, children, and teachers in 10 of the schools in the year prior to intervention commencement [22,23]. –* pg. 3	1. Design intervention with the input of target recipients and delivery staff.
Cao (2015) China	*One important feature of this intervention model was the collaboration between the Education Bureau and Institute of Education, which guaranteed the sustainability of the intervention.* pg. 554	1. Consider collaborating with government departments.<br>2.

Section S5 additional analyses

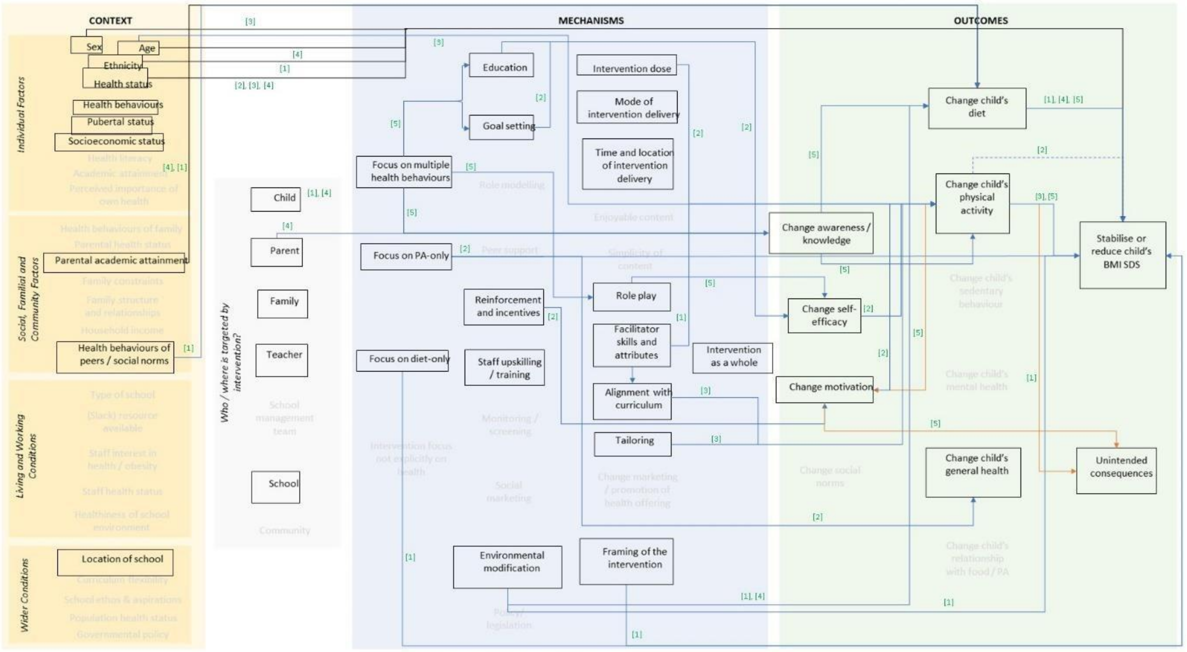

Figure S1. synthesis of effective studies

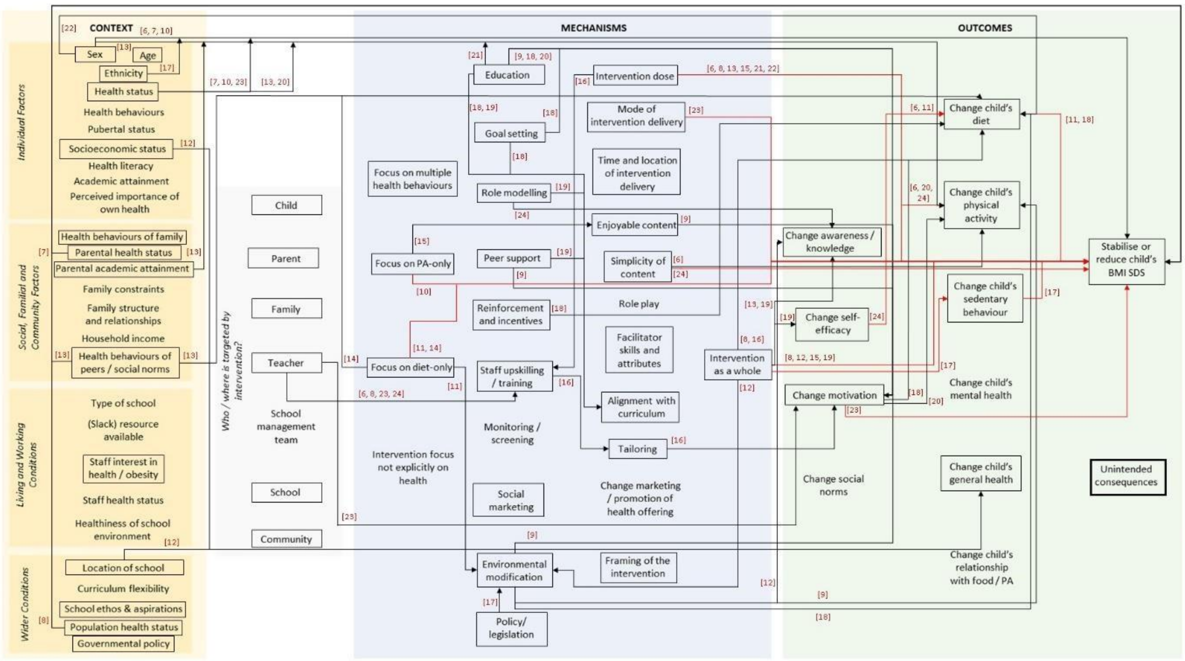

Figure S2. synthesis of ineffective studies

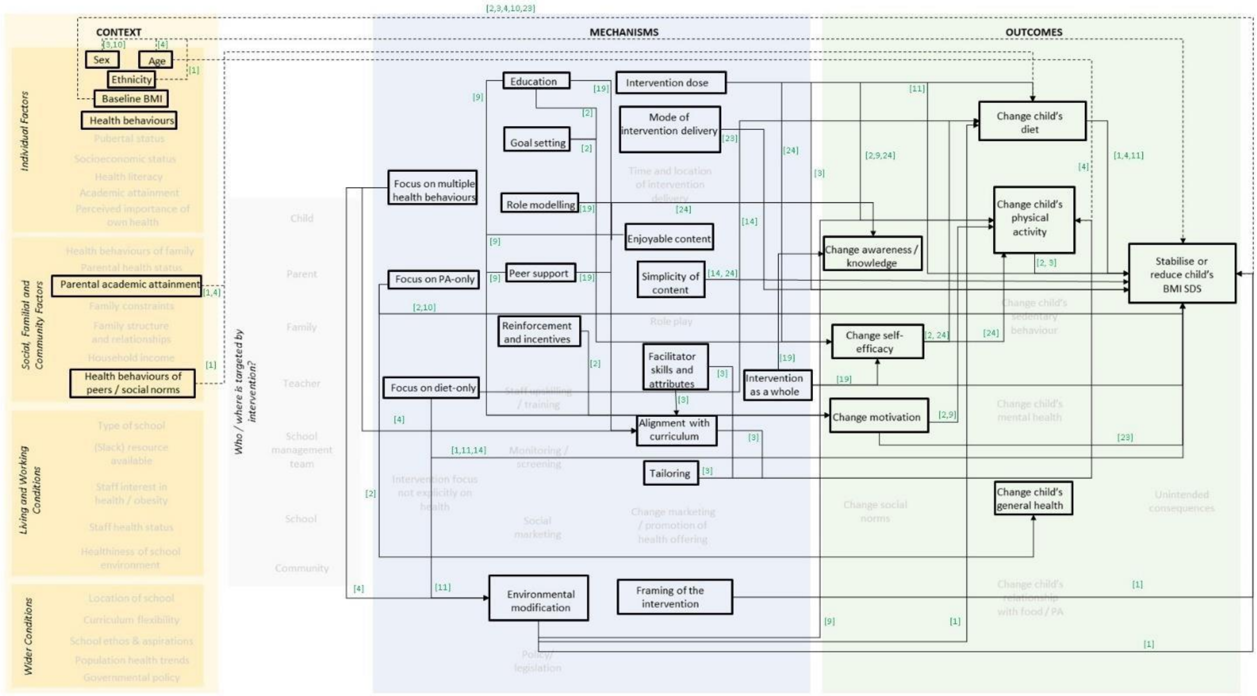

Figure S3. analysis of rigorous studies

Section S6

details of study flow

PRISMA 2009 Flow Diagram

Prevention of obesity in primary school children: Realist review

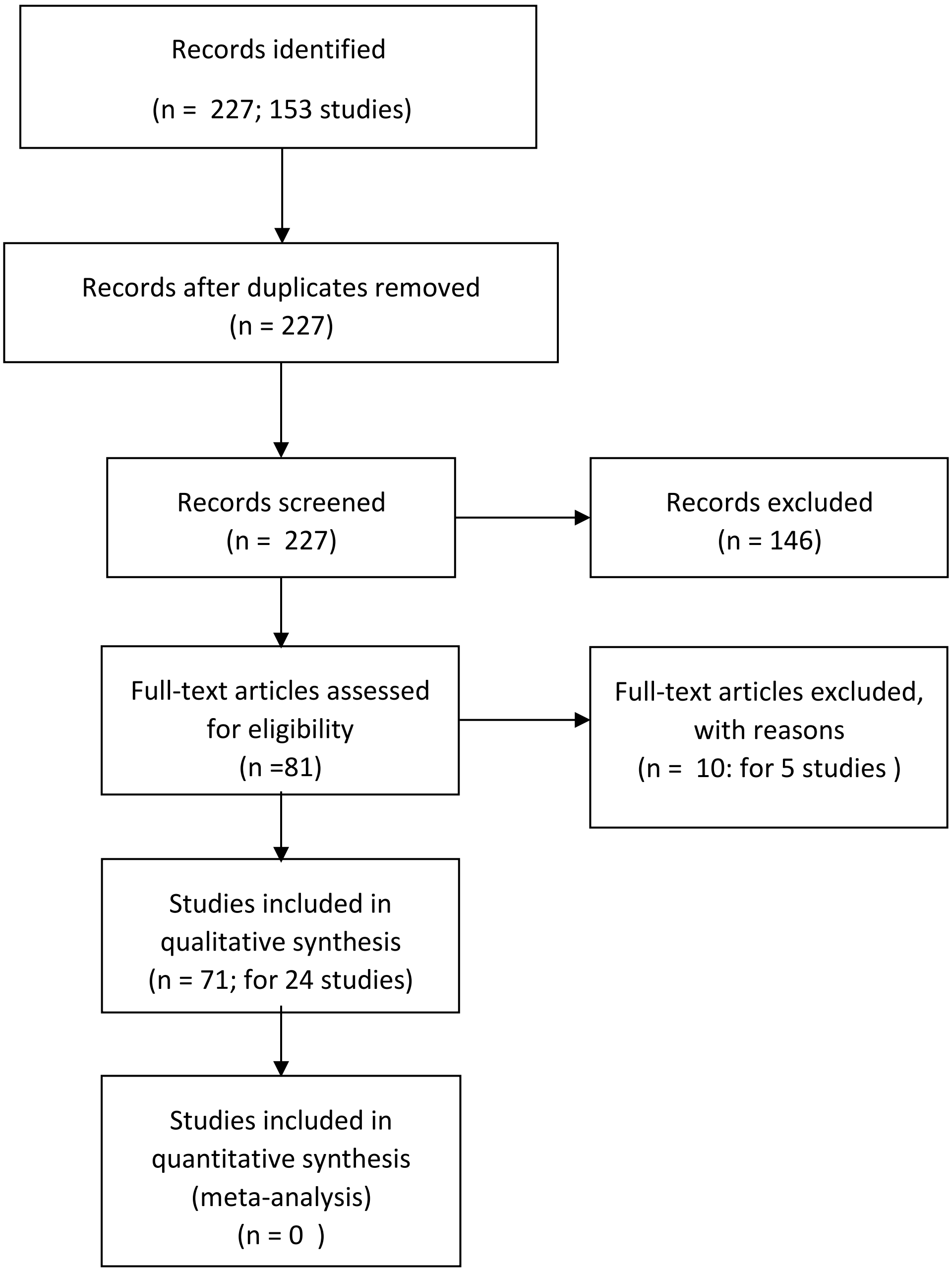

Excluded study table

Study ID	Reason for exclusion	Reference
Haire-Joshu 2010	The intervention setting is outside of school and class and in community only	Haire-Joshu, D. N., M. S. Elliott, M. Davey, C. Caito, N. Loman, D. Brownson, R. (2010). "The use of mentoring programs to improve energy balance behaviors in high-risk children." Obesity **18 Suppl 1**: S75-S83.
Robinson 2010	The intervention setting is outside of school and class and in community only;	Robinson TN, Matheson DM, Kraemer HC, et al. A randomized controlled trial of culturally tailored dance and reducing screen time to prevent weight gain in low-income African American girls: Stanford GEMS. Arch Pediatr Adolesc Med. 2010;164(11):995–1004. doi:10.1001/archpediatrics.2010.197
HEALTHY STUDYGP 2010	Intervention setting is middle school	HEALTHY Study Group. A school-based intervention for diabetes risk reduction. New England journal of medicine 2010;363(5):443-53<br>Hall WJ. School factors as barriers to and facilitators of a preventive intervention for pediatric type 2 diabetes. Translational Behavioral Medicine 2014;4(2):131-40.<br>Marcus MD, Foster GD, Ghormli L. Shifts in BMI category and associated cardiometabolic risk: prospective results from HEALTHY study. Pediatrics 2012;129(4):e983-91.<br>Volpe SL, Hall WJ, Steckler A, Schneider M, Thompson D, Mobley C, et al. Process evaluation results from the HEALTHY nutrition intervention to modify the total school food environment. Health Education Research 2013;28(6):970-8.<br>Schneider M, Hall WJ, Hernandez AE, et al. Rationale, design and methods for process evaluation in the HEALTHY study. International Journal of Obesity (2005). 2009 Aug;33 Suppl 4:S607. DOI: 10.1038/ijo.2009.118.<br>HEALTHY Study Group. HEALTHY study rationale, design and methods: moderating risk of type 2 diabetes in multi-ethnic middle school students.Int J Obes (Lond). 2009 Aug;33 Suppl 4:S4-20. doi: 10.1038/ijo.2009.112.
Amaro 2006	Intervention setting is middle school	Amaro, S., A. Viggiano, A. Di Costanzo, I. Madeo, A. Viggiano, M. E. Baccari, E. Marchitelli, M. Raia, E. Viggiano, S. Deepak, M. Monda and B. De Luca (2006). "Kaledo, a new educational board-game, gives nutritional rudiments and encourages healthy eating in children: a pilot cluster randomized trial." Eur J Pediatr **165**(9): 630-635.
Paineau 2008	Intervention intervention setting is outside of school /class and in community only	Paineau, D. L., F. Beaufils, A. Boulier and et al. (2008). "Family dietary coaching to improve nutritional intakes and body weight control: A randomized controlled trial." Archives of Pediatrics & Adolescent Medicine **162**(1): 34-43.

Included study documents

Study ID, Location (Refs)

1. de Ruyter 2012, Netherlands (1-6)
2. Khan 2014 ,USA (7-9)
3. Li 2010, China (10, 11)
4. Marcus 2009, Sweden, (12)
5. Spiegel 2006,USA,(13)
6. Fairclough 2013, UK, (14-17)
7. Cao 2015 China,(18, 19)
8. Sahota 2001 ,UK, (20, 21)
9. Gutin 2008 USA, (22-24)
10. Lazaar 2007 France, (25)
11. Damsgaard 2014,Denmark ,(26-32)
12. Rush 2012 New Zealand, (33-35)
13. Grydeland 2014 Norway,(36-40)
14. James 2004 ,UK, (41, 42)
15. Meng 2013 China,(43, 44)
16. Rosario 2012 Portugal,(45-47)
17. Foster 2008 , USA,(48)
18. Muckelbauer 2010 Germany,(49-53)
19. Santos 2014 Canada, (54, 55)
20. Siegrist 2013 Germany, (56-58)
21. Williamson 2012 USA, (59-62)
22. Herscovici 2013 Argentina, (63)
23. Johnston 2013,USA,(64)
24. Kipping 2014, UK,(65-71)

References to the 71 documents

1. de Ruyter JC. The effect of sugar‐free versus sugar‐sweetened beverages on satiety, liking and wanting: An 18 month randomized double‐blind trial in children. Study Protocol and amendments. 2013.

2. de Ruyter JC, Katan MB, Kuijper LD, Liem DG, Olthof MR. The effect of sugar-free versus sugar-sweetened beverages on satiety, liking and wanting: an 18 month randomized double-blind trial in children. PLoS One. 2013;8(10):e78039.

3. de Ruyter JC, Olthof MR, Kuijper LD, Katan MB. Effect of sugar-sweetened beverages on body weight in children: design and baseline characteristics of the Double-blind, Randomized INtervention study in Kids. Contemp Clin Trials. 2012;33(1):247-57.

4. de Ruyter JC, Olthof MR, Seidell JC, Katan MB. A trial of sugar-free or sugar-sweetened beverages and body weight in children. N Engl J Med. 2012;367(15):1397-406.

5. Katan MB, de Ruyter JC, Kuijper LD, Chow CC, Hall KD, Olthof MR. Impact of Masked Replacement of Sugar-Sweetened with Sugar-Free Beverages on Body Weight Increases with Initial BMI: Secondary Analysis of Data from an 18 Month Double-Blind Trial in Children. PLoS One. 2016;11(7):e0159771.

6. Olthof MR. A Study of the Effect of Replacing Sugary Drinks by Low-sugar Alternatives on Body Weight and Fat Mass in Children (DRINK). 2009.

7. Hillman CH. ERPS to academics: exercise effects on cognition in school-aged children. Research Portfolio Online Reporting Tools. [Project Funding Information for 2008]. In press 2008.

8. Hillman CH. Exercise Effects on Cognition in School-Aged Children (FITKids). 2011.

9. Khan NA, Raine LB, Drollette ES, Scudder MR, Pontifex MB, Castelli DM, et al. Impact of the FITKids physical activity intervention on adiposity in prepubertal children. Pediatrics. 2014;133(4):e875-83.

10. Li YP, Hu XQ, Schouten EG, Liu AL, Du SM, Li LZ, et al. Report on childhood obesity in China (8): effects and sustainability of physical activity intervention on body composition of Chinese youth. Biomed Environ Sci. 2010;23(3):180-7.

11. Liu A, Hu X, Ma G, Cui Z, Pan Y, Chang S, et al. Evaluation of a classroom-based physical activity promoting programme. Obes Rev. 2008;9 Suppl 1:130-4.

12. Marcus C, Nyberg G, Nordenfelt A, Karpmyr M, Kowalski J, Ekelund U. A 4-year, clusterrandomized, controlled childhood obesity prevention study: STOPP. Int J Obesity. 2009;33(4):408-17.

13. Spiegel SA, Foulk D. Reducing overweight through a multidisciplinary school-based intervention. Obesity. 2006;14(1):88-96.

14. Boddy LM, Knowles ZR, Davies IG, Warburton GL, Mackintosh KA, Houghton L, et al. Using formative research to develop the healthy eating component of the CHANGE! school-based curriculum intervention. BMC Public Health. 2012;12:710.

15. Fairclough SJ. The CHANGE! (Children's Health, Activity and Nutrition: Get Educated!) Project: a clustered randomised controlled trial. 2011.

16. Fairclough SJ, Dumuid D, Mackintosh KA, Stone G, Dagger R, Stratton G, et al. Adiposity, fitness, health-related quality of life and the reallocation of time between children's school day activity behaviours: A compositional data analysis. Prev Med Rep. 2018;11:254-61.

17. Fairclough SJ, Hackett AF, Davies IG, Gobbi R, Mackintosh KA, Warburton GL, et al. Promoting healthy weight in primary school children through physical activity and nutrition education: a pragmatic evaluation of the CHANGE! randomised intervention study. BMC Public Health. 2013;13:626.

18. Cao Z, Wang S, Zheng W, Guo J, Qu S. [Evaluation on the effectiveness of intervention comprehensive program on child obesity, using Generalized Estimating Equation]. Zhonghua Liu Xing Bing Xue Za Zhi. 2014;35(7):773-8.

19. Cao ZJ, Wang SM, Chen Y. A randomized trial of multiple interventions for childhood obesity in China. Am J Prev Med. 2015;48(5):552-60.

20. Sahota P, Rudolf MC, Dixey R, Hill AJ, Barth JH, Cade J. Evaluation of implementation and effect of primary school based intervention to reduce risk factors for obesity. BMJ. 2001;323(7320):1027-9.

21. Sahota P, Rudolf MC, Dixey R, Hill AJ, Barth JH, Cade J. Randomised controlled trial of primary school based intervention to reduce risk factors for obesity. BMJ. 2001;323(7320):1029-32.

22. Gutin B, Yin Z, Johnson M, Barbeau P. Preliminary findings of the effect of a 3-year afterschool physical activity intervention on fitness and body fat: the Medical College of Georgia Fitkid Project. Int J Pediatr Obes. 2008;3 Suppl 1:3-9.

23. Yin Z, Gutin B, Johnson MH, Hanes J, Jr., Moore JB, Cavnar M, et al. An environmental approach to obesity prevention in children: Medical College of Georgia FitKid Project year 1 results. Obes Res. 2005;13(12):2153-61.

24. Yin Z, Hanes J, Jr., Moore JB, Humbles P, Barbeau P, Gutin B. An after-school physical activity program for obesity prevention in children: the Medical College of Georgia FitKid Project. Eval Health Prof. 2005;28(1):67-89.

25. Lazaar N, Aucouturier J, Ratel S, Rance M, Meyer M, Duche P. Effect of physical activity intervention on body composition in young children: influence of body mass index status and gender. Acta Paediatr. 2007;96(9):1315-20.

26. Dalskov SM, Ritz C, Larnkjaer A, Damsgaard CT, Petersen RA, Sorensen LB, et al. The role of leptin and other hormones related to bone metabolism and appetite regulation as determinants of gain in body fat and fat-free mass in 8-11-year-old children. J Clin Endocrinol Metab. 2015;100(3):1196-205.

27. Damsgaard CT, Dalskov SM, Laursen RP, Ritz C, Hjorth MF, Lauritzen L, et al. Provision of healthy school meals does not affect the metabolic syndrome score in 8-11-year-old children, but reduces cardiometabolic risk markers despite increasing waist circumference. Br J Nutr. 2014;112(11):1826-36.

28. Damsgaard CT, Dalskov SM, Petersen RA, Sorensen LB, Molgaard C, Biltoft-Jensen A, et al. Design of the OPUS School Meal Study: a randomised controlled trial assessing the impact of serving school meals based on the New Nordic Diet. Scand J Public Health. 2012;40(8):693-703.

29. Sorensen LB, Dyssegaard CB, Damsgaard CT, Petersen RA, Dalskov SM, Hjorth MF, et al. The effects of Nordic school meals on concentration and school performance in 8- to 11-year-old children in the OPUS School Meal Study: a cluster-randomised, controlled, cross-over trial. Br J Nutr. 2015;113(8):1280-91.

30. Damsgaard ea. suplementary data document 1. Provision of healthy school meals does not affect the metabolic syndrome score in 8–11-year-old children, but reduces cardiometabolic risk markers despite increasing waist circumference. 2014.

31. Damsgaard ea. suplementary data document 2. Provision of healthy school meals does not affect the metabolic syndrome score in 8–11-year-old children, but reduces cardiometabolic risk markers despite increasing waist circumference. 2014.

32. Astrup A. OPUS School Meal Study. https://clinicaltrialsgov/2011.

33. Graham D. Project Energize. 2010.

34. Graham D, Appleton S, Rush E, McLennan S, Reed P, Simmons D. Increasing activity and improving nutrition through a schools-based programme: Project Energize. 1. Design, programme, randomisation and evaluation methodology. Public Health Nutr. 2008;11(10):1076-84.

35. Rush E, Reed P, McLennan S, Coppinger T, Simmons D, Graham D. A school-based obesity control programme: Project Energize. Two-year outcomes. Br J Nutr. 2012;107(4):581-7.

36. Bergh IH, Bjelland M, Grydeland M, Lien N, Andersen LF, Klepp KI, et al. Mid-way and postintervention effects on potential determinants of physical activity and sedentary behavior, results of the HEIA study - a multi-component school-based randomized trial. Int J Behav Nutr Phys Act. 2012;9:63.

37. Bjelland M, Bergh IH, Grydeland M, Klepp KI, Andersen LF, Anderssen SA, et al. Changes in adolescents' intake of sugar-sweetened beverages and sedentary behaviour: results at 8 month midway assessment of the HEIA study--a comprehensive, multi-component school-based randomized trial. Int J Behav Nutr Phys Act. 2011;8:63.

38. Bjelland M, Hausken SE, Bergh IH, Grydeland M, Klepp KI, Andersen LF, et al. Changes in adolescents' and parents' intakes of sugar-sweetened beverages, fruit and vegetables after 20 months: results from the HEIA study - a comprehensive, multi-component school-based randomized trial. Food Nutr Res. 2015;59:25932.

39. Grydeland M, Bergh IH, Bjelland M, Lien N, Andersen LF, Ommundsen Y, et al. Intervention effects on physical activity: the HEIA study - a cluster randomized controlled trial. Int J Behav Nutr Phys Act. 2013;10:17.

40. Lien N, Bjelland M, Bergh IH, Grydeland M, Anderssen SA, Ommundsen Y, et al. Design of a 20-month comprehensive, multicomponent school-based randomised trial to promote

healthy weight development among 11-13 year olds: The HEalth In Adolescents study. Scand J Public Health. 2010;38(5 Suppl):38-51.

41. James J, Thomas P, Cavan D, Kerr D. Preventing childhood obesity by reducing consumption of carbonated drinks: cluster randomised controlled trial. 2004.

42. James J, Thomas P, Kerr D. Preventing childhood obesity: two year follow-up results from the Christchurch obesity prevention programme in schools (CHOPPS). 2007;335(7623):762.

43. Ma G. Study on comprehensive prevention and control technology of childhood obesity based on dietary nutrition. 2009.

44. Meng L, Xu H, Liu A, van Raaij J, Bemelmans W, Hu X, et al. The costs and cost-effectiveness of a school-based comprehensive intervention study on childhood obesity in China. PLoS One. 2013;8(10):e77971.

45. Rosario R, Araujo A, Oliveira B, Padrao P, Lopes O, Teixeira V, et al. Impact of an intervention through teachers to prevent consumption of low nutrition, energy-dense foods and beverages: A randomized trial. Prev Med. 2013;57(1):20-5.

46. Rosario R, Oliveira B, Araujo A, Lopes O, Padrao P, Moreira A, et al. The impact of an intervention taught by trained teachers on childhood overweight. Int J Environ Res Public Health. 2012;9(4):1355-67.

47. University of Minho. The Impact of an Intervention Taught by Trained Teachers on Childhood BMI z Score. 2011.

48. Foster GD, Sherman S, Borradaile KE, Grundy KM, Vander Veur SS, Nachmani J, et al. A policy-based school intervention to prevent overweight and obesity. Pediatrics. 2008;121(4):e794802.

49. Muckelbauer R, Libuda L, Clausen K, Kersting M. Long-term process evaluation of a schoolbased programme for overweight prevention. Child Care Health Dev. 2009;35(6):851-7.

50. Muckelbauer R, Libuda L, Clausen K, Reinehr T, Kersting M. A simple dietary intervention in the school setting decreased incidence of overweight in children. Obes Facts. 2009;2(5):282-5.

51. Muckelbauer R, Libuda L, Clausen K, Toschke AM, Reinehr T, Kersting M. Promotion and provision of drinking water in schools for overweight prevention: randomized, controlled cluster trial. Pediatrics. 2009;123(4):e661-7.

52. Muckelbauer R, Libuda L, Clausen K, Toschke AM, Reinehr T, Kersting M. Immigrational background affects the effectiveness of a school-based overweight prevention program promoting water consumption. Obesity (Silver Spring). 2010;18(3):528-34.

53. Research Institute of Child Nutrition Dortmund. Promoting Water Consumption for Prevention of Overweight in School Children in a Controlled Intervention Trial (trinkfit). 2007.

54. Santos RG, Durksen A, Rabbanni R, Chanoine JP, Lamboo Miln A, Mayer T, et al. Effectiveness of peer-based healthy living lesson plans on anthropometric measures and physical activity in elementary school students: a cluster randomized trial. JAMA Pediatr. 2014;168(4):330-7.

55. University of Manitoba. Healthy Buddies Manitoba. https://clinicaltrialsgov/2013.

56. Siegrist M, Hanssen H, Lammel C, Haller B, Halle M. A cluster randomised school-based lifestyle intervention programme for the prevention of childhood obesity and related early cardiovascular disease (JuvenTUM 3). BMC Public Health. 2011;11:258.

57. Siegrist M, Lammel C, Haller B, Christle J, Halle M. Effects of a physical education program on physical activity, fitness, and health in children: the JuvenTUM project. Scand J Med Sci Sports. 2013;23(3):323-30.

58. Technische Universität München. School Based Health Promotion Program in Secondary Schools (JuvenTUM 3). https://clinicaltrialsgov/2009.

59. Martin C. The Louisiana (LA) Health Project. https://clinicaltrialsgov/2006.

60. Newton RL, Thomson JL, Rau KK, Ragusa SA, Sample AD, Singleton NN, et al. Psychometric characteristics of process evaluation measures for a rural school-based childhood obesity prevention study: Louisiana Health. Am J Health Promot. 2011;25(6):417-21.

61. Williamson DA, Champagne CM, Harsha D, Han H, Martin CK, Newton R, Jr., et al. Louisiana (LA) Health: design and methods for a childhood obesity prevention program in rural schools. Contemp Clin Trials. 2008;29(5):783-95.

62. Williamson DA, Champagne CM, Harsha DW, Han H, Martin CK, Newton RL, Jr., et al. Effect of an environmental school-based obesity prevention program on changes in body fat and body weight: a randomized trial. Obesity (Silver Spring). 2012;20(8):1653-61.

63. Herscovici CR, Kovalskys I, De Gregorio MJ. Gender differences and a school-based obesity prevention program in Argentina: a randomized trial. Rev Panam Salud Publica. 2013;34(2):75-82.

64. Johnston CA, Moreno JP, El-Mubasher A, Gallagher M, Tyler C, Woehler D. Impact of a school-based pediatric obesity prevention program facilitated by health professionals. J Sch Health. 2013;83(3):171-81.

65. Campbell R, Rawlins E, Wells S, Kipping RR, Chittleborough CR, Peters TJ, et al. Intervention fidelity in a school-based diet and physical activity intervention in the UK: Active for Life Year 5. Int J Behav Nutr Phys Act. 2015;12:141.

66. Kipping RR, Howe LD, Jago R, Campbell R, Wells S, Chittleborough CR, et al. Effect of intervention aimed at increasing physical activity, reducing sedentary behaviour, and increasing fruit and vegetable consumption in children: active for Life Year 5 (AFLY5) school based cluster randomised controlled trial. BMJ. 2014;348:g3256.

67. Lawlor DA. Active for Life Year 5. 2010.

68. Lawlor DA, Howe LD, Anderson EL, Kipping RR, Campbell R, Wells S, et al. The Active for Life Year 5 (AFLY5) school-based cluster randomised controlled trial: effect on potential mediators. BMC Public Health. 2016;16:68.

69. Lawlor DA, Jago R, Noble SM, Chittleborough CR, Campbell R, Mytton J, et al. The Active for Life Year 5 (AFLY5) school based cluster randomised controlled trial: study protocol for a randomized controlled trial. Trials. 2011;12:181.

70. Lawlor DA, Kipping RR, Anderson EL, Howe LD, Chittleborough CR, Moure-Fernandez A, et al. Active for Life Year 5: a cluster randomised controlled trial of a primary school-based intervention to increase levels of physical activity, decrease sedentary behaviour and improve diet. Public Health Research. 4. Southampton (UK)2016.

71. Lawlor DA, Peters TJ, Howe LD, Noble SM, Kipping RR, Jago R. The Active for Life Year 5(AFLY5) school-based cluster randomised controlled trial protocol: detailed statistical analysis plan. Trials. 2013;14:234.
